# Antibiotic dispensation rates among participants in community-driven health research projects in Arctic Canada

**DOI:** 10.1186/s12889-019-7193-3

**Published:** 2019-07-15

**Authors:** Kathleen Williams, Amy Colquhoun, Rachel Munday, Karen J. Goodman, CANHelp Working Group

**Affiliations:** 1 School of Public Health, University of Alberta, 7-142 Katz Building, 87 Ave – 114 St, Edmonton, AB T6G 2E1 Canada; 2 Analytics and Performance Reporting, Alberta Health, Edmonton, AB Canada; 3 Susie Husky Health Centre, Aklavik, NT Canada; 4 Department of Medicine, University of Alberta, Edmonton, AB Canada

**Keywords:** *Helicobacter pylori*, Antibiotic exposure, Antibiotic resistance, Treatment failure, Arctic Canada

## Abstract

**Background:**

Community-driven projects that aim to address public concerns about health risks from *H. pylori* infection in Indigenous Arctic communities (estimated *H. pylori* prevalence = 64%) show frequent failure of treatment to eliminate the bacterium. Among project participants, treatment effectiveness is reduced by antibiotic resistance of infecting *H. pylori* strains, which in turn, is associated with frequent exposure to antibiotics used to treat other infections. This analysis compares antibiotic dispensation rates in Canadian Arctic communities to rates in urban and rural populations in Alberta, a southern Canadian province.

**Methods:**

Project staff collected antibiotic exposure histories for 297 participants enrolled during 2007–2012 in Aklavik, Tuktoyaktuk, and Fort McPherson in the Northwest Territories, and Old Crow, Yukon. Medical chart reviews collected data on systemic antibiotic dispensations for the 5-year period before enrolment for each participant. Antibiotic dispensation data for urban Edmonton, Alberta (average population ~ 860,000) and rural northern Alberta (average population ~ 450,000) during 2010–2013 were obtained from the Alberta Government Interactive Health Data Application.

**Results:**

Antibiotic dispensation rates, estimated as dispensations/person-years (95% confidence interval) were: in Arctic communities, 0.89 (0.84, 0.94); in Edmonton, 0.55 (0.55, 0.56); in rural northern Alberta, 0.63 (0.62, 0.63). Antibiotic dispensation rates were higher in women and older age groups in all regions. In all regions, the highest dispensation rates occurred for β-lactam and macrolide antibiotic classes.

**Conclusions:**

These results show more frequent antibiotic dispensation in Arctic communities relative to an urban and rural southern Canadian population.

## Background

Antibiotic-resistant bacteria are a major public health concern because they limit the effectiveness of therapeutic options available for the treatment of bacterial infections. Much evidence suggests that exposure to antibiotics leads to antibiotic-resistant bacterial infections. In particular, consistent evidence shows an association between frequent exposure to antibiotics for the treatment of unrelated bacterial infections and the prevalence of antibiotic-resistant *Helicobacter pylori* infection [1–6]*.*

The Canadian North *Helicobacter pylori* (CAN*Help*) Working Group links northern Canadian communities, health care providers and regional health officials with University of Alberta investigators to conduct community-driven research aimed at addressing concerns about health risks from *H. pylori* infection; the ultimate goal is to inform public health policy pertaining to control of *H. pylori* infection [7]. In Canada, northern Indigenous communities are disproportionately burdened by *H. pylori* infection compared to multi-ethnic populations in southern Canada [8–10]. Available data show stomach cancer rates to be elevated in Indigenous Arctic populations as well, [11–13] an occurrence of great concern to affected communities and their health care providers.

*H. pylori* is a bacterium that colonizes the lining of the stomach and/or duodenum, [14] where it is nearly always accompanied by gastritis. This infection often persists indefinitely; in persistent cases, it increases the risk of peptic ulcer disease and stomach cancer [15–18]. Treatment to eliminate *H. pylori* infection has been observed to improve peptic ulcer healing, decrease peptic ulcer recurrence and reduce the risk of stomach cancer [19, 20]. Frequent treatment failure in populations with high prevalence of *H. pylori* infection is a major impediment to the development of effective *H. pylori* control strategies for such populations [21]. CAN*Help* community projects collected data on participants’ exposure to antibiotics as part of inquiry aimed at identifying causes of treatment failure.

Few studies have collected information on antibiotic exposure in geographically defined communities, even though it is known that the frequency of antibiotic use varies by geographic region, as well as other factors including age and sex [22–24]. Geographic differences in prescribing practices or non-prescription access to antibiotics likely contribute to differences in the occurrence of antibiotic-resistant infections across geographic regions [25, 26].

The aims of the current study are to describe antibiotic dispensation rates, as a measure of the frequency of overall exposure to systemic antibiotics, by community, sex, age and antibiotic class, in Canadian Arctic communities and compare these rates to those of urban and rural outpatient populations in the southern Canadian province of Alberta. These results will shed light on whether increased exposure to antibiotics is a potential explanation for reduced effectiveness of antibiotics in Indigenous Arctic populations, thus making them vulnerable to poor infection outcomes.

## Methods

This analysis includes participants in community *H. pylori* projects conducted by the CAN*Help* Working Group in 4 hamlets in the western Arctic region of Canada: projects launched in Aklavik, Northwest Territories (NT) in 2007, Old Crow, Yukon (YT) in 2010, Tuktoyaktuk (NT) in 2011, and Fort McPherson (NT) in 2012 [27–38]. Each of these communities is predominantly Indigenous, mainly Gwich’in (Athabaskan First Nations) or Inuvialuit (western Canadian Inuit): Aklavik had 594 residents (2006 census), with 92% identifying as either Inuvialuit or Gwich’in; Old Crow had 245 residents (2011 census), with 90% identifying as Vuntut Gwitchin; Tuktoyaktuk had 854 residents (2011 census), with 92% identifying as Inuvialuit; and Fort McPherson had 792 residents (2011 census, with 94% identifying as Indigenous (mainly Gwich’in) [39, 40]. Each community project, guided by a planning committee comprising community leaders, included several components: surveys to collect risk factor data; non-invasive screening for *H. pylori* infection; endoscopy with gastric biopsy for pathological and microbiological assessment; treatment; and ongoing knowledge exchange activities. Diverse outreach strategies sought to encourage all community residents to participate. Project staff obtained informed consent from participants 17 years of age or older, parental consent for children under 17 years of age, and assent from 7 to 16-year-old participants.

The lead author (KW) collected chart review data for her MSc thesis research on factors associated with antibiotic-resistant *H. pylori* infection; [41] specifically, she collected antibiotic exposure history pertaining to CAN*Help* community project participants who fulfilled either or both of the following criteria for inclusion in this analysis: 1) available *H. pylori* isolates cultured from gastric biopsies and tested for antibiotic susceptibility; 2) completed treatment to eliminate *H. pylori* infection and were tested after treatment to assess post-treatment infection status. We collected each participant’s antibiotic exposure history from their medical chart, housed in community health centres, using a chart review tool to record systemic antibiotics prescribed for any reason during the 5 years before the participant’s enrolment date. This 5-year exposure period spanned different calendar years across communities because community projects were launched sequentially: our chart review recorded medications prescribed primarily during 2002–2007 in Aklavik, 2005–2010 in Old Crow, 2006–2011 in Tuktoyaktuk, and 2007–2012 in Fort McPherson (a few participants enrolled after the launch year, so their antibiotic exposure period began slightly later). Data collected from medical charts included: demographic factors; frequency of antibiotic prescriptions; types of antibiotics prescribed; and the reason for each prescription. An antibiotic prescription was defined as a single prescription of at least one systemic antibiotic regardless of the dose, dosing frequency or duration of the prescription.

As a measure of antibiotic use frequency, we estimated antibiotic dispensation rates, defining dispensation as the process by which a prescribed medication is given to a patient for whom a prescription was written. We used prescriptions as proxies for dispensations because medical charts noted prescriptions rather than dispensations, and because prescriptions given to patients at health centres in participating northern communities are dispensed routinely from locally stocked medication by health centre staff at the time of prescription; thus, in this study population, it can be assumed that each prescription noted in a medical chart was dispensed.

We expressed dispensation rates as the average of the number of antibiotic courses dispensed per person per year during each participant’s 5-year review period. We calculated these rates by dividing the total number of systemic antibiotic courses prescribed for all participants during the 5-year review period by the product of the number of participants and the sum of the number of years reviewed in the medical chart of each participant. We estimated antibiotic dispensation rates by community (Aklavik, Old Crow, Fort McPherson, Tuktoyaktuk), sex, age (categorized in 20-year age groups), and antibiotic class (β-lactams, macrolides, nitroimidazoles, nitrofurans, fluoroquinolones, tetracyclines, rifamycins).

To put the antibiotic dispensation rates observed in the western Arctic communities in perspective, we compared them to rates observed in Alberta, a western Canadian province located directly south of the Northwest Territories. We estimated antibiotic dispensation rates for the outpatient populations of urban Edmonton (Alberta’s capital) and rural northern Alberta using the online Alberta Government Interactive Health Data Application (IHDA), [42] which incorporates data from the following sources: the Pharmaceutical Information Network (PIN) database; the Alberta Health Care Insurance Plan Adjusted Mid-Year Population Registry Files; and the Alberta Health and Wellness Postal Code Translation File. The PIN captures all drug dispensation events occurring in community pharmacies across Alberta. Aggregate PIN data are available through the IHDA by geography, age group, sex, year, and antibiotic class. We restricted estimates of the Edmonton antibiotic dispensation rate to the population residing within the city limits (subzones Z4.1-Z4.4) and the rural northern Alberta rate to residents of the Alberta North Zone (subzones Z5.1-Z5.5); when we accessed data for both regions in March 2017, the only available data covered 2010 through 2013.

We estimated the outpatient antibiotic dispensation rates by dividing the number of antibiotic courses dispensed by the sum of the population during each year from 2010 through 2013. For statistical comparison, we used an estimation approach, presenting 95% confidence intervals (CI) for all estimated rates, rather than statistical significance testing, following best practice in statistical methods for epidemiology [43, 44]. To compare the Arctic community population to each of the two Alberta populations, we estimated rate differences and 95% CIs.

## Results

We collected antibiotic exposure histories from medical charts of CAN*Help* community project participants: 164 from Aklavik; 67 from Old Crow; 52 from Fort McPherson; and 14 from Tuktoyaktuk. Table 1 presents the demographic characteristics of the study population.

**Table 1:** Demographic characteristics of study population

	n	Age Distribution		Proportion Female %
		Age Group^a^ (years)	%	
Four Arctic Communities	**297**	4–19 20–39 40–59 60–81	12.8 30.0 41.1 16.2	54.9
Aklavik	164	4–19 20–39 40–59 60–80	18.3 34.8 34.8 12.2	51.1
Old Crow	67	5–19 20–39 40–59 60–77	7.5 32.8 43.3 16.4	52.2
Tuktoyaktuk	14	6–19 20–39 40–59 60–78	7.1 14.3 50.0 28.6	71.4
Fort McPherson	52	15–19 20–39 40–59 60–81	3.9 15.4 55.8 25.0	59.6
Edmonton Outpatients^b^	~ 865,000	0–19 20–39 40–59 60–90+	22.5 33.6 27.9 16.1	49.5
Rural Northern Alberta Outpatients^c^	~ 460,000	0–19 20–39 40–59 60–90+	27.8 31.7 27.5 13.0	47.8

^a^Age category labels for Edmonton and rural Alberta outpatients do not indicate the minimum and maximum age values; age categories labels for the upper and lower age groups vary across communities to indicate the minimum and maximum age values

^b^Alberta Government Interactive Health Data Application Edmonton City Centre population including both sexes and all ages ranged from 830,213 to 903,256 during 2010–2013

^c^Alberta Government Interactive Health Data Application North Zone population including both sexes and all ages ranged from 440,406 to 476,886 during 2010–2013

Boldface data are set to differentiate the values in the total row from values in other rows, which represent subsets of the total

Among the 297 participants, the median number of systemic antibiotic prescriptions during the 5-year review period was 3 (IQR, 1–6; range, 0–38; mean, 4.3; SD, 4.6).

Table 2 presents estimated antibiotic dispensation rates in CAN*Help* community project participants and the outpatient populations of Edmonton and rural northern Alberta. The overall antibiotic dispensation rate was notably higher in the CAN*Help* community project study population (8.9 courses per 10 person-years) than in northern rural Alberta (6.3 courses per 10 person-years) or Edmonton (5.5 courses per 10 person-years). The estimated antibiotic dispensation rate in western Arctic communities was 3.3 (95% CI: 2.9, 3.8) courses per 10 person-years higher than the Edmonton outpatient population rate and 2.6 (95% CI: 21, 31) courses per 10 person-years higher than the rural northern Alberta outpatient rate.

**Table 2:** Estimated antibiotic dispensation rates in western Arctic communities, Edmonton and rural northern Alberta

	Antibiotic Courses Dispensed	
	per person-year	95% CI
Four Arctic Communities	**0.89**	**0.84, 0.94**
Aklavik	0.84	0.78, 0.91
Old Crow	0.90	0.80, 1.01
Fort McPherson & Tuktoyaktuk	0.99	0.89, 1.11
Edmonton Outpatients	**0.55**	**0.55, 0.56**
Rural Northern Alberta Outpatients	**0.63**	**0.62, 0.63**

Boldface data are set to differentiate the values in the total row from values in other rows, which represent subsets of the total

Northern Alberta is a large geographic region with two small cities and vast sparsely populated areas. Table 3 presents estimated antibiotic dispensation rates for sub-zones of this region. Of note, the two cities have antibiotic dispensation rates slightly lower than Edmonton’s, while rates in the sparely populations zones range from 5.9 to 7.3 per 10 person-years, all substantially lower than the estimated rates in the Arctic communities.

**Table 3:** Estimated antibiotic dispensation rates in the rural northern Alberta sub-zones

Northern Alberta Subzone	Population during 2010–2013^a^		Antibiotic Courses Dispensed	
	Minimum	Maximum	per/Person/Year	95% CI
Total	**440,406**	**476,886**	**0.63**	**0.62, 0.63**
SW	92,750	95,976	0.61	0.60, 0.61
SE	83,677	87,998	0.71	0.71, 0.72
Central West	36,836	43,090	0.59	0.58, 0.59
NW	92,752	95,229	0.73	0.73, 0.73
NE	4761	5007	0.66	0.65, 0.67
Fort McMurray (city)	65,457	79,553	0.53	0.52, 0.53
Grande Prairie (city)	64,007	70,034	0.52	0.52, 0.53

^a^Alberta Government Interactive Health Data Application North Zone population including both sexes and all ages during 2010–2013

Boldface data are set to differentiate the values in the total row from values in other rows, which represent subsets of the total

Table 4 presents estimated antibiotic dispensation rates for the Arctic communities and the outpatient populations of Edmonton and rural northern Alberta by sex, age and antibiotic class. Across all populations, the estimated dispensation rate was higher in woman than in men, the highest rates occurred in senior adults, and β-lactam antibiotics were dispensed at a substantially higher rate than other antibiotic classes. Stratification by age group and sex shows that dispensation rates were substantially higher in the Arctic communities within each age group and in both sexes. Of note, compared to the Alberta populations, dispensation rates in the Arctic communities were similar for β-lactams and macrolides, lower for fluoroquinolones and tetracyclines, higher for nitrofurans, and substantially higher for nitroimidazoles.

**Table 4:** Estimated antibiotic dispensation rates in western Arctic communities, Edmonton and rural northern Alberta by sex, age and antibiotic class

	Western Arctic Communities			Edmonton†		Rural Northern Alberta‡	
	n	Rate/ Person/Year	95% CI	Rate/ Person/Year	95% CI	Rate/ Person/Year	95% CI
Total Population	**297**	**0.89**	**0.84, 0.94**	**0.555**	**0.554, 0.556**	**0.625**	**0.624, 0.627**
Sex							
Female	163	1.09	1.02, 1.17	0.662	0.661, 0.664	0.772	0.770, 0.774
Male	134	0.64	0.58, 0.71	0.450	0.449, 0.451	0.491	0.489, 0.492
Age Group							
< 20 years	38	0.75	0.63, 0.89	0.493	0.492, 0.495	0.626	0.624, 0.629
20–39 years	89	0.88	0.79, 0.97	0.464	0.462, 0.465	0.543	0.541, 0.544
40–59 years	122	0.85	0.78, 0.93	0.591	0.590, 0.593	0.606	0.604, 0.608
≥ 60 years	48	1.11	0.98, 1.26	0.768	0.765, 0.770	0.862	0.859, 0.866
Antibiotic Class							
β-lactams	178	0.31	0.28, 0.33	0.259	0.258, 0.259	0.312	0.311, 0.313
Macrolides	100	0.11	0.09, 0.13	0.097	0.097, 0.097	0.107	0.107, 0.108
Nitroimidazoles	60	0.06	0.05, 0.07	0.001	0.001, 0.001	0.001	0.001, 0.001
Nitrofurans	22	0.04	0.03, 0.05	0.023	0.023, 0.023	0.024	0.024, 0.024
Fluoroquinolones	29	0.03	0.03, 0.04	0.077	0.077, 0.077	0.081	0.080, 0.081
Tetracyclines	16	0.02	0.01, 0.02	0.043	0.043, 0.043	0.036	0.036, 0.036
Rifamycins	1	0.0007	0.000, 0.004	§	–	§	–

†Alberta Government Interactive Health Data Application Edmonton City Centre population including both sexes and all ages during 2010–2013

‡Alberta Government Interactive Health Data Application North Zone population including both sexes and all ages during 2010–2013

§Rifamycin antibiotic usage was not reported in the IHDA dataset

Boldface data are set to differentiate the values in the total row from values in other rows, which represent subsets of the total

## Discussion

Evidence from this study suggests that antibiotics are dispensed more frequently to western Arctic Canadians than to either urban or rural residents of the southwestern Canadian province of Alberta, independently of age and sex. This study showed additionally that antibiotics were dispensed more frequently to women relative to men and to the elderly relative to other age groups in each of the 3 settings investigated. Dispensation rates for the most frequently dispensed antibiotic classes, β-lactams and macrolides, was similar in the 3 populations, while fluoroquinolones and tetracyclines were dispensed less frequently and nitrofurans and nitroimidazoles were dispensed more frequently in the Arctic communities.

In the present study, the dispensation rate for systemic antibiotic prescriptions among participants in CAN*Help* Working Group community projects was 8.9 prescriptions per 10 person-years; an even higher antibiotic prescription rate of 15 per 10 person-years was estimated for an Alaska Native population in 2003 [2]. The urban antibiotic dispensation rates we estimated for Edmonton and the two small northern Alberta cities ranged from 5.2–5.5 per 10 person-years; a slightly lower antibiotic dispensation rate of 4.4 per 10 person-years was estimated from a 1992 study of United States physician practices [45]. More frequent use of antibiotics in rural and remote Arctic regions of North America relative to urban settings in this region is compatible with diverse explanations including higher incidence of infectious diseases, more limited access to diagnostic technology used to confirm bacterial infections leading to more frequent dispensation of antibiotics without a confirmed diagnosis, and different prescription practices resulting from more frequent provision of health care by nurses in phone consultation with physicians rather than by physicians directly [46, 47].

Variation in the use of antibiotics for the treatment of common bacterial infections may contribute to variation in antibiotic-resistant infection across geographic regions and sociodemographic groups [25, 26]. For example, the frequency of clarithromycin-resistant *H. pylori* infection has been reported to be higher in children than in adults, with evidence suggesting that this contrast is due to more frequent use of macrolide antibiotics in children for the treatment of respiratory tract infections [1, 48]. The frequency of metronidazole-resistant *H. pylori* infection has been reported to be higher in women than in men, [49] perhaps due to widespread use of nitroimidazole antibiotics for the treatment of gynaecological infections [2, 49–54]. For example, in a study conducted in the United Kingdom, nitroimidazole antibiotics were prescribed more frequently to women than to men, and the prevalence of metronidazole-resistant *H. pylori* infection was also higher in women relative to men [4]. Similarly, reports of higher prevalence of tetracycline-resistant *H. pylori* infection in women relative to men may be due to more frequent use of tetracycline in women for the treatment of urogenital infections [55]. Higher prevalence of fluoroquinolone-resistant *H. pylori* infection in women relative to men and in youth relative to adults may be associated with more frequent use of fluoroquinolone antibiotics for the treatment of urogenital and respiratory tract infections, respectively [55].

An important limitation of the present analysis is the potential underestimation of antibiotic use among participants in the Arctic Canadian community *H. pylori* projects. Participants’ medical charts only capture antibiotics dispensed at their community health centre. Many residents of the project communities spend time away from home and may receive health care in other locations. Most residents of northern Canadian communities, however, receive most of their health care at their community health centre; it is, therefore, likely that most antibiotics dispensed during the review period for this study were captured by our chart review. The data sources used for the Alberta populations would similarly miss prescriptions dispensed outside Alberta; however, given that Albertans are all entitled to health care provided by the provincial government, they are not likely to seek outpatient care for infections outside the province.

Another limitation pertains to our differential strategy for capturing dispensation events across territorial and provincial populations. In the northern communities included here, prescription events equate with dispensations. In Alberta, however, primary nonadherence is possible: an individual may be prescribed a drug but may not fill the prescription, precluding its dispensation [56, 57]. While this is unlikely to impact our ability to assess differences in dispensation patterns, it does limit our ability to investigate plausible explanations for results. Similarly, we were unable to assess whether a person who was dispensed an antibiotic used the medication as prescribed. As a result, we are restricted to reporting differences in potential exposure to antibiotics across these populations.

In this study, the time periods for which antibiotic dispensation rates were estimated differ in each of the Arctic communities (2002–2007, 2005–2010, 2006–2011, 2007–2012) due to the sequential conduct of these projects. These periods overlap to different degrees with the time period captured by the publicly accessible Alberta data, which was 2010–2013 at the time of data analysis. We would not, however, expect substantial changes in antibiotic dispensation during 2002 to 2013 based on studies of outpatient populations in Ontario, Canada and in the United States, which suggest that antibiotic dispensation rates remained relatively stable across age groups during this time period [58, 59].

## Conclusions

This study reveals more frequent dispensation of antibiotics in Arctic Canada relative to urban and rural populations in southern Canada. These results suggest that increased exposure to antibiotics is a potential explanation for reduced effectiveness of antibiotics in Indigenous Arctic populations for treating infections such as *H. pylori* infection. More generally, Indigenous Arctic populations may be particularly vulnerable to antibiotic-resistant infections associated with frequent exposure to antibiotics. The evidence generated by this analysis will be useful for developing strategies aimed at reducing health disparities arising from inequitable health care in Indigenous Arctic populations relative to other North American populations.

## Data Availability

Antibiotic dispensation data collected from community project participants are not available through open access due to terms stipulated in community-university partnership agreements. Data requests are reviewed according to a process outlined in the Statement on Stewardship and Dissemination of Knowledge Generated Collaboratively in CAN*Help* Working Group Community Projects available at http://canhelpworkinggroup.ca/research-program/collaborative-partnerships/. To initiate the data request process, contact the corresponding author. The interactive health data application (IHDA) dataset analysed for the current study are available at the following url: www.ahw.gov.ab.ca/IHDA_Retrieval/.

## Abbreviations

CAN*Help* Working Group: Canadian North *Helicobacter pylori* Working Group
CI: Confidence interval
*H. pylori*: *Helicobacter pylori*
IHDA: Alberta Interactive Health Data Application
IQR: Interquartile Range
NT: Northwest Territories
PIN: Pharmaceutical Information Network
PPI: Proton-pump inhibitor
SD: Standard deviation
YT: Yukon Territory
